# Individual, behavioural and home environmental factors associated with eating behaviours in young adolescents

**DOI:** 10.1016/j.appet.2017.01.001

**Published:** 2017-05-01

**Authors:** Natalie Pearson, Paula Griffiths, Stuart J.H. Biddle, Julie P. Johnston, Emma Haycraft

**Affiliations:** a School of Sport, Exercise & Health Sciences, Loughborough University, UK; b Institute of Sport, Exercise & Active Living (ISEAL), Victoria University, Australia; c Department of Sport Science, School of Science and Technology, Nottingham Trent University, UK

**Keywords:** Eating, Home environment, Adolescents, Parents, Energy-dense snacks, Fruit, Vegetables

## Abstract

This study aimed to examine individual, behavioural and home environmental factors associated with frequency of consumption of fruit, vegetables and energy-dense snacks among adolescents. Adolescents aged 11–12 years (n = 521, 48% boys) completed a paper-based questionnaire during class-time which included a Food Frequency Questionnaire assessing their consumption of fruit, vegetables, and energy-dense (ED) snacks, and items assessing habits, self-efficacy, eating at the television (TV), eating with parents, parenting practices, and home availability and accessibility of foods. Multiple linear regression analyses showed that eating fruit and vegetables while watching TV and home availability and accessibility of fruit and vegetables were positively associated with frequency of fruit consumption and vegetable consumption, while home accessibility of ED snack foods was negatively associated with frequency of fruit consumption. Habit for eating ED snack foods in front the TV, eating ED snack foods while watching TV, and home availability of ED snacks were positively associated with frequency of ED snack consumption. This study has highlighted the importance of a healthy home environment for promoting fruit and vegetable intake in early adolescents and also suggests that, if snacking while TV viewing occurs, this could be a good opportunity for promoting fruit and vegetable intake. These findings are likely to be useful for supporting the development of multi-faceted interventions and aid us in knowing what advice to give to parents to help them to help their young adolescents to develop and maintain healthy eating habits.

## 1 Background

Adolescence is a significant developmental life stage where health behaviours are often established and become habitual. Unhealthy eating behaviours including snacking on energy-dense foods and low intakes of fruit and vegetables are particularly common characteristics of many adolescents' diets, and have a significant impact on both immediate and long term physiological and mental health conditions including obesity indicators (Piernas & Popkin, 2010), cancers (Colditz and Frazier, 1995, Maynard et al., 2003), and mental health disorders (Jacka et al., 2011, Jacka et al., 2013). Eating behaviours and habits developed during adolescence tend to persist into adulthood (Craigie, Lake, Kelly, Adamson, & Mathers, 2011), and thus decreasing the consumption of energy-dense foods and increasing the consumption of fruits and vegetables during adolescence are important targets for nutrition interventions. Identifying potentially modifiable factors of adolescent eating behaviours is imperative for the design of successful interventions. Furthermore, identifying eating behaviours that share modifiable factors is potentially useful as eating behaviours do not occur in isolation and such data could underpin dietary interventions aiming to change multiple eating behaviours.

Many potential correlates of adolescent eating behaviours have been identified. For example, review level evidence suggests that habit can determine food choices and eating behaviours (Reinaerts et al., 2007, van't Riet et al., 2011), and that repeated (habitual) food choices and eating behaviours are often associated with environmental cues – e.g., coming home from work or school (Neal, Wood, Labrecque, & Lally, 2012). Self-efficacy is another correlate of eating behaviour, with evidence suggesting that higher levels of self-efficacy, that is, feeling confident in one's ability to successfully undertake a task, are related to health behaviour changes such as healthier eating behaviours (Pearson et al., 2011a, Pearson et al., 2011b).

Although adolescence is associated with increased autonomy, parents still typically provide foods for children and are responsible for mealtimes (Neumark-Sztainer, Larson, Fulkerson, Eisenberg, & Story, 2010). Eating meals as a family and parental role modelling have both been associated with healthier adolescent eating behaviours (Fink et al., 2014, Gillman et al., 2000). Availability and accessibility of foods are powerful predictors of consumption, with greater availability and accessibility of fruits and vegetables being related to greater intake in children and adolescents (Cook et al., 2015, Loth et al., 2016, Pearson et al., 2009). Furthermore, not making unhealthy foods available or accessible, i.e. employing covert restriction, has been linked to lower intake of such foods (Ogden, Reynolds, & Smith, 2006).

Food-related parenting practices are commonly used by parents of adolescents (Loth, MacLehose, Fulkerson, Crow, & Neumark-Sztainer, 2013) and also relate to adolescents' eating behaviours. Pressure to eat certain foods, or finish meals, has been associated with lower consumption of healthy pressured foods (e.g., soup (Galloway, Fiorito, Francis, & Birch, 2006)) but greater consumption of unhealthy foods (e.g., unhealthy snacks (Brown & Ogden, 2004);) in children and to less healthy eating attitudes and behaviours (Haycraft, Goodwin, & Meyer, 2014) and greater weight (Loth et al., 2013) in adolescents. Restriction of foods can be associated with greater subsequent intake, particularly if the restriction has been overt (e.g., “No, you can't have another biscuit”) or if food has been used as a reward (Birch & Fisher, 1998). Parental use of restriction has also been linked to higher adolescent weight (Loth et al., 2013).

Other health behaviours have been found to play an important role in determining eating behaviours. Behaviours – such as watching television whilst eating – have been related to increased food consumption (Blass et al., 2006, Pearson et al., 2011a, Pearson et al., 2011b) which, in turn, can lead to weight gain. For example, adolescents who watch TV whilst eating meals have been found to have less healthy diets than those who do not watch TV whilst eating meals (Feldman, Eisenberg, Neumark-Sztainer, & Story, 2007) and TV viewing has been linked to greater unhealthy snack food consumption in children and adolescents (Gebremariam et al., 2013, Pearson and Biddle, 2011).

While numerous factors have been identified as impacting eating behaviours, it is unlikely that these exert their effects individually. Given that theoretically based nutrition interventions have been shown to be more effective than those without a theoretical underpinning (Cerin et al., 2009, Glanz and Bishop, 2010), behavioural theories should be utilised to provide a framework for studying factors associated with eating behaviours. There is support for the use of social–ecological models in understanding health behaviours (Golden & Earp, 2012). These posit that factors at the individual (e.g. habits), social (parental modelling) and physical (e.g. availability of foods at home) environmental levels interact to influence health behaviour (McLeroy et al., 1988, Sallis et al., 2008). Few studies have examined the influence of correlates across multiple levels, and/or have examined the same correlates for multiple eating behaviours, both of which are likely to be beneficial for the development of multifaceted interventions to promote healthy eating. Furthermore, where studies have examined correlates at multiple levels of the social-ecological model, it is typical that factors significant in a univariate model are entered into a multivariate model regardless of their ‘level’. We are unaware of any study that has examined the effect of correlates of multiple eating behaviours at each level separately (e.g. factors significant at the individual level all entered into a multivariate model to determine the contribution of each factor at the individual level) before combining into one model. Such information is important for providing modifiable determinants to target in a multi-level intervention. Using a social–ecological framework, the present study aimed to examine individual, behavioural and home environmental factors associated with the frequency of consumption of fruit, vegetables and energy-dense snacks among young adolescents aged 11–12 years.

## 2 Methods

### 2.1 Study procedure and participants

Cross-sectional data were collected between May 2013 and June 2014. Study procedures were approved by the Ethical Advisory Committee of the host university. Data were obtained from young adolescents in their first year (Year 7) of secondary school (aged 11–12 years) recruited from four secondary schools in the East Midlands region of the UK. All students in Year 7 of participating schools were eligible and received an information leaflet to take home for a parent or guardian with details of the study (n = 683). Under existing ethical guidelines, it was necessary to seek consent from parents for each child's participation, and no information could be accessed regarding characteristics of non-respondents. Adolescent participants provided assent before completing written questionnaires during class time. In total, 562 pupils provided parental consent (82% response rate) and 521 were present on the data collection days and completed the questionnaire (76% response rate).

### 2.2 Measures

Participants completed paper-based questionnaires during a school lesson under the supervision of trained researchers and class teachers. Participants provided their date of birth and gender.

#### 2.2.1 Eating behaviours

Food intake was assessed using a Food Frequency Questionnaire (FFQ). This FFQ was based on previously validated indices of food intake (Rockett et al., 1997) but options were reduced to focus on the specific foods of interest (namely, fruit, vegetables, and energy-dense snacks) and assessed intake frequency during the past week. Students indicated how frequently they consumed eighteen food items during a usual week. Seven response categories ranged from ‘never’ to ‘more than three a day’. The frequency of consumption of the eighteen food items in the past month was converted to a daily equivalent, which is an established method (Willett, 1998, Neumark-Sztainer et al., 2003, Pearson et al., 2011a, Pearson et al., 2011b). Daily equivalents were calculated as follows: never (0·00 per d); one-two days a week (0·2 per d); 3–4 days a week (0·5 per d); five-six days a week (0·7 per d); once a day (1.0 per d); twice a day (2.0 per d); three or more a day (3.0 per d). The daily intake of fruit, vegetables, and energy-dense snacks was calculated by summing the daily equivalents for the food items in each food group. The estimated daily intake of ‘fruit’ included the summed equivalence of five fruit items (apples, bananas, oranges, grapes and other fruit), the daily equivalent of ‘vegetables’ included the summed equivalence of five vegetable items (carrots, peas, broccoli, salad and other vegetables), the daily equivalence of ‘energy-dense snacks’ included the summed equivalence of eight snack food items (potato crisps/potato chips, snack crackers, sweets (candy), chocolate, chocolate biscuits, regular biscuits, muffins/cakes, cereal bars).

#### 2.2.2 Individual, behavioural, social and physical environmental factors

##### 2.2.2.1 Individual

Adolescents were asked four questions about their habits for eating snack foods in front of the television using the previously validated Self-Report Behavioural Automaticity Index (SRBAI) (Gardner, Abraham, Lally, & de Bruijn, 2012): ‘eating snack foods (e.g. chocolate/biscuits/crisps) while watching television (TV) is something I do automatically’; ‘ … without having to remember’; ‘ … without thinking’; ‘ … before I realise I'm doing it’. They were asked the same four questions regarding eating fruit and vegetables in front of the television. Response options were given on a five-point Likert scale, ranging from (1) ‘strongly disagree’ to (5) ‘strongly agree’. Responses were summed separately to provide two habit scores, one for eating snacks in front of the TV (Cronbach's α = 0.86) and one for eating fruit and vegetables in front of the TV (Cronbach's α = 0.91).

Based on a previously used scale (Pearson et al., 2011a, Pearson et al., 2011b), adolescents were asked six questions about how confident that would feel about reducing their energy-dense snack food consumption (i.e. snacks including chocolate, crisps, biscuits, sweets (candy)): ‘How sure are you that you could not eat snack foods when you're with your friends'; ‘ … you're with your family’; ‘ … after school’; ‘ … when you're alone’; ‘ … when you're bored’; ‘ … when you're feeling down’. They were asked the same six questions about not eating snack foods in front of the television and about eating more fruit and vegetables. Response options were given on a five-point Likert scale, ranging from (1) ‘Not at all sure’ to (5) ‘very sure’. Responses were summed separately to provide three self-efficacy scores, one for not eating energy-dense snacks (Cronbach's α = 0.89), one for not eating energy-dense snacks in front of the TV (Cronbach's α = 0.88), and one for eating more fruit and vegetables (Cronbach's α = 0.90).

##### 2.2.2.2 Behavioural

Adolescents were asked how often they ate breakfast, lunch, dinner, energy-dense snacks and fruit and vegetables while also watching the television during a typical week using a previously used questionnaire by Matheson et al. (Matheson, Killen, Wang, Varady, & Robinson, 2004). Response options were given on a four-point Likert scale ranging from (1) ‘Never’ to (4) ‘Every day’. The frequency of consumption of the meals and snacks while watching TV was converted to a daily equivalent. Daily equivalents were calculated as follows: never (0·00 per d); one-two days a week (0·2 per d); 3–6 days a week (0·6 per d); everyday (1.0 per d).

##### 2.2.2.3 Social environmental

Adolescents were asked how often, during a typical week, they ate the following with their parents: breakfast, dinner, breakfast in front of the TV, dinner in front of the TV, and snacks in front of the TV. Response options were given on a five-point Likert scale ranging from (1) ‘Never/less than once a week’ to (5) ‘Every day’. The frequency of consumption of the meals and snacks with parents was converted to a daily equivalent. Daily equivalents were calculated as follows: never (0·00 per d); once a week (0.14); two-three times a week (0·36 per d); 4–6 days times a week (0·6 per d); everyday (1.0 per d).

Adolescents were asked questions regarding perceptions of parental pressure to eat, food restriction, and food as a reward using items from the Kid's Child Feeding Questionnaire (KCFQ) (Carper et al., 2000, Kaur et al., 2006). For all items, response options were given on a three-point Likert scale: (1) ‘No’, (2) ‘sometimes’, (3) ‘Yes’. Adolescents were asked to answer all questions about the parent/caregiver who is typically responsible for feeding them/providing meals. Adolescents were asked seven questions regarding pressure to eat (e.g. ‘If you say, “I'm not hungry” at dinnertime, does your parent say, “You need to eat anyway”?’). Scores of the seven items were summed and divided by seven to create the ‘pressure to eat’ score (Cronbach's α = 0.73). Adolescents were asked seven questions regarding parental restriction (e.g. ‘Does your parent every say things like “you've had enough to eat now, you need to stop”?’). Scores of the seven items were summed and divided by seven to create the ‘restriction’ score (Cronbach's α = 0.71). Adolescents were asked two questions regarding parental use of food as a reward (e.g. ‘My parents let me have snacks (e.g. sweets/chocolates) as a reward for good behaviour’). Scores of the two items were summed and divided by two to create the ‘food as a reward’ score (Cronbach's α = 0.75).

##### 2.2.2.4 Physical environmental

Adolescents were asked four questions regarding availability of energy-dense snacks in the home in the past week (e.g. ‘how frequently were the following items available to you at home last week’: cakes/biscuits, crisps, chocolates, sweets), and two questions regarding the availability of fruit and vegetables (fruit and vegetables). Response options were given on a four-point Likert scale ranging from (1) ‘Never/rarely’ to (4) ‘Always’. Scores of the four energy-dense snacks were summed to create the ‘home availability of energy-dense snacks’ score (Cronbach's α = 0.84) and scores of the fruit and vegetables were summed to create the ‘home availability of fruit and vegetables’ score (Cronbach's α = 0.83).

Adolescents were asked two questions regarding the accessibility of energy-dense snacks in the home and four questions regarding accessibility of fruit and vegetables in the past week (e.g. ‘in the past week, were there any fruits that were prepared and ready for you to eat as part of a meal or snack?’). Response options were given on a three-point Likert scale ranging from (1) ‘No, never’ to (3) ‘Yes, always’. Scores of the two energy-dense snacks questions were summed to create the ‘home accessibility of energy-dense snacks’ score (Cronbach's α = 0.71) and scores of the four fruit and vegetable questions were summed to create the ‘home accessibility of fruit and vegetables’ score (Cronbach's α = 0.70).

### 2.3 Statistical analysis

All analyses were conducted using the SPSS statistical software package 22.0 (SPSS Inc., Chicago, IL, USA). Descriptive statistics were used to summarise the demographic and eating characteristics of the sample. Independent t tests were conducted to determine gender differences in all variables.

Unadjusted linear regression analyses (model 1) were conducted to examine associations between the proposed individual, social and physical environmental factors and the eating behaviours of interest (fruit, vegetable, and energy-dense snack consumption). As suggested by Bursac et al. (Bursac, Gauss, Williams, & Hosmer, 2008), a p value of 0.25 was used to identify variables significant in Model 1. All individual factors that were significantly associated with the eating behaviour in the unadjusted analyses (p ≤ 0.25) were subsequently entered into multiple linear regression models (model 2). All behavioural factors that were significantly associated (p ≤ 0.25) with the eating behaviour in the unadjusted analyses were entered into multiple linear regression models (model 3). All social environmental factors that were significantly associated (p ≤ 0.25) with the eating behaviour in the unadjusted analyses were entered into multiple linear regression models (model 4). All home environmental factors that were significantly associated (p ≤ 0.25) with the eating behaviour in the unadjusted analyses were entered into multiple linear regression models (model 5). Finally, all variables that were significantly associated with eating behaviours in model 2, 3, 4, and 5 (p ≤ 0.05) were entered into fully adjusted multiple linear regression models (model 6). Multicolinearity was assessed for each model using the tolerance and variance inflation factors. Tolerance inflation values were always greater than 0.1 and variance inflation values ranged from 1 to 1.3 (not presented but available on request from the corresponding author).

All analyses were conducted separately for each eating behaviour (fruit, vegetable, and energy-dense snack consumption), and controlled for gender and age in each model.

## 3 Results

### 3.1 Sample characteristics

Just over half of the adolescent sample was female (52%) and the mean age of the adolescents was 11.64 (SD 0.48) years. Table 1 presents the means and standard deviations of individual, behavioural, social and physical environmental variables, and dietary behaviours by gender. Boys ate breakfast in front of the TV with their parents more frequently than girls (p < 0.05), and reported slightly higher perceptions of parental pressure to eat (p < 0.01). Boys also reported eating fruit and energy-dense snacks more frequently than girls (p < 0.05).

### 3.2 Associations between individual, behavioural, social and physical environmental factors and adolescent eating behaviours

#### 3.2.1 Individual factors (model 2)

After adjusting for gender and age, five individual factors were significantly associated with fruit consumption (model 1, Table 2), five with vegetable consumption (model 1, Table 3), and four with ED snack consumption (model 1, Table 4), respectively. After adjusting for all significant variables from model 1 (for each eating behaviour separately), habit for eating snack foods while watching TV was negatively associated with fruit consumption and habit for eating fruit and vegetables while watching TV was positively associated with fruit consumption (model 2, Table 2). Habit for eating snack foods while watching TV was negatively associated with vegetable consumption, while habit for eating fruit and vegetables while watching TV and self-efficacy for increasing fruit and vegetable consumption were positively associated with vegetable consumption (model 2, Table 3). Habit for eating snack foods while watching TV was positively associated with energy-dense snack consumption (model 2, Table 4), and this remained significant in model 6 (Table 4).

#### 3.2.2 Behavioural factors (model 3)

After adjusting for gender and age, three behavioural factor was significantly associated with fruit consumption (model 1, Table 2), four with vegetable consumption (model 1, Table 3) and five with energy-dense snack consumption respectively (model 1, Table 4). After adjusting for all significant variables from model 1 (for each eating behaviour separately), eating fruit and vegetables while watching TV was positively associated with fruit consumption and vegetable consumption (model 3, Table 2, Table 3) while eating dinner while watching TV and eating energy-dense snacks while watching TV were negatively associated with both fruit and vegetable consumption. Eating fruit and vegetables whilst watching TV remained significantly positively associated with both fruit and vegetable consumption in model 6 (Table 2, Table 3), and eating energy-dense snacks while watching TV remained negatively associated with fruit consumption in model 6 (Table 2). After adjusting for all significant behavioural variables from model 1, only eating energy-dense snacks while watching TV was positively associated with energy-dense snack consumption (model 3, Table 4), and this remained significant in model 6 (Table 4).

#### 3.2.3 Social environmental factors (model 4)

After adjusting for gender and age, six social environmental factors were significantly associated with fruit consumption (model 1, Table 2), five with vegetable consumption (model 1, Table 3), and six with energy-dense snack food consumption (model 1, Table 4).

After adjusting for all significant social variables from model 1 (for each eating behaviour separately), eating breakfast in front of the TV at home together with parents and receiving food as a reward were positively associated with fruit consumption, while eating snacks in front of the TV together with parents and parental food restriction were negatively associated with fruit consumption (model 4, Table 2). Eating breakfast in front of the TV at home together with parents and receiving food as a reward remained positively associated with fruit consumption in model 6 (Table 2). Eating dinner at home together with parents was positively associated with vegetable consumption, while parental food restriction was negatively associated with vegetable consumption (model 4, Table 3). Eating snacks in front of the TV together with parents and parental food restriction were positively associated with energy-dense snack consumption (model 4, Table 4). No social environmental factor remained significant in model 6 for vegetable or energy-dense snack food consumption.

#### 3.2.4 Physical environmental factors (model 5)

After adjusting for gender and age, all four physical environmental factors were significantly associated with fruit (model 1, Table 2), vegetable (model 1, Table 3), and energy-dense snack consumption, respectively (model 1, Table 4). After adjusting for all significant physical environmental variables from model 1 (for each eating behaviour separately), home availability of fruit and vegetables, and home accessibility of fruit and vegetables were positively associated with fruit consumption (model 5, Table 2). Home availability of fruit and vegetables remained significant in model 6 (Table 2). Home availability of energy-dense snacks was negatively associated and home availability of fruit and vegetables was positively associated with vegetable consumption (model 5, Table 3). Availability of fruit and vegetables remained positively associated with vegetable consumption in model 6 (Table 3). Home availability and home accessibility of energy-dense snacks were positively associated with energy-dense snack consumption, while home availability of fruit and vegetables was negatively associated with energy-dense snack food consumption (model 5, Table 4). Home availability of energy-dense snacks remained positively associated with energy-dense snack food consumption in model 6 (Table 4).

## 4 Discussion

The aim of this study was to examine individual, behavioural and home environmental factors associated with frequency of consumption of fruit, vegetables and energy-dense snacks among young adolescents. While most factors were differentially associated with food consumption in adolescents, eating in front of the television and home availability and accessibility of foods appeared to be consistently associated with fruit, vegetable and energy-dense snack food consumption. Eating fruit and vegetables whilst watching TV were positively associated with frequency of both fruit and vegetable consumption, and eating energy-dense snacks while watching TV was positively associated with frequency of ED snack consumption.

Eating fruit and vegetables in front of the television predicted adolescents' consumption of both fruits and vegetables, while habitually eating ED snack foods in front of the television predicted their ED food intake. Such findings highlight the strong associations between television viewing and food intake but interestingly suggest TV viewing can be related to both healthy and unhealthy snacking behaviours. This is in contrast with review level evidence which suggests that TV viewing is associated with *lower* fruit and vegetable intake (Hobbs et al., 2015, Pearson and Biddle, 2011) but may indicate that if children are going to snack in front of the TV, then snacking on fruit and vegetables in front of the screen is one way to potentially increase children's intake of these foods. We also found that eating ED snacks whilst watching TV and having a habit for doing so were both linked to greater ED snack food intake. TV viewing has been shown to be associated with mindless eating, and paying less attention to hunger and fullness cues (Wansink, 2004), and so it is possible that this notion applies regardless of whether the food is healthy (fruit and vegetables) or unhealthy (ED snacks). These findings highlight potentially modifiable behavioural factors which predict food intake and which may be useful targets in future interventions.

We also found physical environmental factors – specifically home availability and accessibility – to predict increased consumption. Interestingly, greater availability and accessibility of fruit and vegetables, alongside less accessibility of ED snack foods, predicted greater fruit consumption in these adolescents. Increased availability of fruit and vegetables at home predicted higher intake of vegetables too. Such findings corroborate previous literature (Cook et al., 2015, Pearson et al., 2009), but extend it by also considering the role of accessibility and availability of ED snack foods as predictors of healthy food intake. Unsurprisingly, home availability of ED snack foods was a strong predictor of ED snack food intake, further reinforcing the importance of the physical environment in determining adolescents' eating behaviours. Covert restriction – i.e. not having the food available/accessible - has been suggested as an effective way to limit intake of certain foods (Ogden et al., 2006) and this message may need reinforcing to parents to support healthy eating practices. Interventions with children have successfully shown that increasing the provision of fruits and vegetables at home can have immediate and sustained increases in consumption of these foods (Wyse, Wolfenden, & Bisquera, 2015). Our findings have important implications for future healthy eating interventions with adolescents as they reinforce the importance of availability and accessibility for promoting adolescents' healthy food intake.

While individual factors (habit, self-efficacy) and social environment factors (eating with parents, parenting practices) were all significantly associated with fruit and/or vegetable consumption in some of the models, these factors were not significant in model 6, suggesting that other factors – particularly the physical environment – are more powerful determinants of fruit and vegetable intake in adolescents and explain the associations observed with the individual and social environmental factors. Habit did not predict intake of fruit and vegetables but did predict ED snack consumption. It may be that the palatable and rewarding nature of ED snack foods helps to establish this habitual behaviour, and that the lack of rewarding properties in fruits and vegetables makes habit formation less likely in this age group, particularly if other options are available. Self-efficacy may be a more effective determinant of snack food choice in conjunction with suggested health behaviour changes, where it has previously been found to be effective at promoting healthier behaviours (Lawlor et al., 2016, Pearson et al., 2011a, Pearson et al., 2011b). Parents are still influential in their adolescent child's eating behaviours yet eating meals as a family, and the use of feeding practices like pressure or restriction, seem to be less important predictors of adolescents' consumption. This highlights the overriding significance of the physical environment in impacting young adolescents' eating behaviours.

The results of this study have implications for health promotion and preventing the development of overweight/obesity in adolescents. Our findings reinforce the importance of parents creating a healthy home environment in order to promote healthy eating behaviours in their adolescents. Given that home availability of both healthy and unhealthy foods was associated with both healthy and unhealthy snack food consumption respectively, and that snacking on healthy and or unhealthy foods in front of the TV was associated with greater consumption, the dual approach of increasing the availability/accessibility of healthy snack foods as well as reducing the availability/accessibility of unhealthy snack foods could be one avenue for future studies to explore as a way to improve adolescent dietary behaviours and subsequent weight status. More needs to be done to promote covert restriction of ED snack foods, and to help parents to help their adolescents develop habits for healthy snacking in front of the screens (if screens are going to be viewed) as a way to increase their daily fruit and vegetable intake and maintain a healthy weight status.

Strengths of this study include consideration of a wide range of variables (individual, behavioural and home environmental factors), the large sample size and the ability to determine the relative influence of factors at multiple levels individually and combined. Limitations include the cross-sectional design, the small convenience sample, and the fact that we only collected data on consumption of certain fruits, vegetables and energy-dense snacks. While it is possible that other foods in these groups were consumed by the participants, we were keen to minimise participant burden to ensure that the data we collected were as accurate and reliable as possible. The results are also limited by the use of self-reports, which may not be wholly accurate in these young adolescents, and by the fact that we did not obtain any height/weight data on our participants which prevented the exploration of any weight status differences in adolescents' eating behaviours and the predictors of.

In conclusion, this novel study has used a social–ecological framework to consider a combination of individual, behavioural and home environmental factors associated with the frequency of consumption of fruit, vegetables and energy-dense snacks among young adolescents. Notable findings include highlighting the importance of a healthy home environment for promoting fruit and vegetable intake in young adolescents. The findings also indicate that, if TV viewing occurs (and particularly snacking at the TV), having fruit and vegetables available and accessible for snacking at the TV can be an effective way to increase children's intake of these healthy foods. These findings are likely to be useful for supporting the development of multi-faceted interventions and aid us in knowing what advice to give to parents to help them to help their adolescents to develop and maintain healthy eating habits.

## Financial support

This research was funded by a British Heart Foundation project grant (PG/12/70/29777). The funding body had no role in the analysis or preparation of the manuscript.

## Author contributions

The contribution of authors was as follows: N. P. conceptualised the study, and conducted the study with J.P.J. N.P carried out the statistical analyses and drafted the manuscript. E.H, P.G, and S.J.H.B were involved in the design of the study and contributed to the drafting of the manuscript. All authors read, contributed to and approved the final manuscript.

## Conflict of interest

None.

## Figures and Tables

**Table 1:** Description of individual, behavioural, social and physical environmental variables relating to eating behaviours of adolescent participants aged 11–12 years.

	Total (n = 521)	Boys (n = 248)	Girls (n = 273)
		Mean (SD)	Mean (SD)
**Individual**			
Habit for eating snack foods while watching TV (range 1–5)	2.97 (1.01)	2.93 (1.04)	3.01 (0.99)
Habit for eating fruit and vegetables while watching TV (range 1–5)	2.79 (1.01)	2.81 (1.08)	2.77 (0.95)
Self-efficacy for not eating snack foods when watching TV/DVD's (range 1–5)	2.98 (1.07)	3.02 (1.15)	2.94 (1.00)
Self-efficacy for increasing fruit and vegetable consumption (range 1–5)	2.89 (1.06)	2.83 (1.15)	2.93 (0.96)
Self-efficacy for reducing energy-dense snack food consumption (range 1–5)	2.78 (1.05)	2.80 (1.11)	2.76 (1.00)
**Behavioural (frequency/day)**			
Eating breakfast while watching TV	0.52 (0.43)	0.59 (0.44)	0.46 (0.42)
Eating lunch while watching TV	0.54 (0.43)	0.57 (0.44)	0.51 (0.42)
Eating dinner while watching TV	0.63 (0.43)	0.63 (0.44)	0.62 (0.43)
Eating fruit and vegetables while watching TV	0.44 (0.36)	0.47 (0.37)	0.42 (0.35)
Eating energy-dense snacks while watching TV	0.50 (0.34)	0.48 (0.34)	0.51 (0.34)
**Social environment**			
Eating breakfast at home together with parents (frequency/day)	0.36 (0.40)	0.38 (0.41)	0.34 (0.39)
Eating dinner at home together with parents (frequency/day)	0.74 (0.34)	0.74 (0.35)	0.73 (0.34)
Eating dinner in front of the TV with parents (frequency/day)	0.33 (0.38)	0.31 (0.38)	0.35 (0.37)
Eating breakfast in front of the TV with parents (frequency/day)	0.20 (0.34)	0.24 (0.37)	0.17 (0.30)*
Eating snacks in front of the TV with parents (frequency/day)	0.35 (0.36)	0.36 (0.37)	0.33 (0.35)
Parental pressure to eat (Range 1–3)	1.79 (0.46)	1.86 (0.47)	1.74 (0.45)**
Parental food restriction (Range 1–3)	2.46 (0.36)	2.45 (0.36)	2.46 (0.36)
Food as a reward (Range 1–3)	1.89 (0.71)	1.96 (0.73)	1.84 (0.69)
**Physical environment**			
Home availability of energy-dense snack foods (range 4–16)	9.60 (2.85)	9.51 (2.70)	9.66 (2.99)
Home availability of fruit and vegetables (range 2–8)	5.79 (1.84)	5.64 (1.91)	5.91 (1.78)
Home accessibility of energy-dense snack foods (range 2–6)	4.03 (1.17)	4.09 (1.18)	3.97 (1.15)
Home accessibility of fruit and vegetables (range 4–12)	9.06 (1.92)	8.96 (1.95)	9.16 (1.89)
**Eating behaviours (frequency/day)**			
Fruit	1.97 (1.59)	2.02 (1.69)	1.92 (1.49)*
Vegetables	1.80 (1.45)	1.78 (1.44)	1.82 (1.45)
Energy-dense snack foods	3.77 (3.07)	3.93 (3.31)	3.61 (2.83)*

Mean values were significantly different from those of males *p < 0.05, **p < 0.01, ***p < 0.001.

**Table 2:** Regression coefficients and 95% CIs of adjusted and unadjusted multiple linear regression analyses: individual, behavioural, social and physical environmental variables and (Model 1) child fruit consumption adjusted for child gender and age; (Model 2) child fruit consumption adjusted for child gender and age and all ‘individual’ variables significant in the unadjusted linear regression analyses; (Model 3) child fruit consumption adjusted for child gender and age and all ‘behavioural’ variables significant in the unadjusted linear regression analyses; (Model 4) child fruit consumption adjusted for child gender and age and all ‘social environmental’ variables significant in the unadjusted linear regression analyses; (Model 5) child fruit consumption adjusted for child gender and age and all ‘physical environmental’ variables significant in the unadjusted linear regression analyses; (Model 6) child fruit consumption adjusted for child gender and age and all variables significant in Model 2–5.

Individual	Fruit (frequency/day)		Fruit (frequency/day)		Fruit (frequency/day)	
	Unstandardised regression coefficient (95% CI)	*P*	Unstandardised regression coefficient (95% CI)	*P*	Unstandardised regression coefficient (95% CI)	*P*
	Model 1		Model 2		Model 6	
Habit for eating snack foods while watching TV	0.19 (0.10, 0.47)	**0.193**	−0.19 (-0.35, −0.04)	**0.020**	0.01 (-0.17, 0.19)	0.929
Habit for eating fruit and vegetables while watching TV	−0.29 (-0.57, −0.01)	**0.041**	0.30 (0.14, 0.45)	**0.000**	0.12 (-0.14, 0.28)	0.142
Self-efficacy for not eating snack foods when watching TV/DVDs	−0.32 (-0.61, −0.03)	**0.030**	0.05 (-0.12, 0.22)	0.542		
Self-efficacy for increasing fruit and vegetable consumption	0.15 (0.02, 0.29)	**0.023**	0.09 (-0.06, 0.23)	0.247		
Self-efficacy for reducing energy-dense snack food consumption	0.11 (-0.03,0.24)	**0.115**	0.01 (-0.17, 0.17)	0.956		
**Behavioural**	**Model 1**		**Model 3**		**Model 6**	
Eating breakfast while watching TV	0.08 (-0.24, 0.40)	0.624				
Eating lunch while watching TV	−0.13 (-0.45, 0.19)	0.418				
Eating dinner while watching TV	−0.28 (-0.60, 0.04)	**0.098**	−0.38 (-0.71, 0.05)	0.023	−0.37 (-0.74, 0.01)	0.052
Eating fruit and vegetables while watching TV	1.08 (0.70, 1.45)	**0.000**	1.38 (0.99, 1.77)	**0.000**	1.07 (0.59,1.54)	**0.000**
Eating energy-dense snacks while watching TV	−0.38 (-0.77, 0.01)	**0.059**	−0.60 (-1.01, 0.19)	0.004	−0.60 (-1.12, 0.08)	0.023
**Social environment**	**Model 1**		**Model 4**		**Model 6**	
Eating breakfast at home together with parents	0.08 (-0.26, 0.42)	0.655				
Eating dinner at home together with parents	0.35 (-0.05, 0.75)	**0.086**	0.21 (-0.22, 0.65)	0.326		
Eating dinner in front of the TV with parents	0.13 (-0.35, 0.38)	0.945				
Eating breakfast in front of the TV with parents	0.24 (-0.17, 0.65)	**0.246**	0.53 (0.06, 0.99)	**0.020**	0.58 (0.09, 1.05)	**0.018**
Eating snacks in front of the TV with parents	−0.37 (-0.75, 0.01)	**0.06**	−0.62 (-1.07, −0.18)	**0.006**	−0.26 (-0.73, 0.22)	0.285
Parental pressure to eat	0.36 (0.05, 0.67)	**0.02**	0.10 (-0.25, 0.45)	0.57		
Parental food restriction	−0.37 (-0.077, 0.03)	**0.07**	−0.47 (-0.91, −0.03)	**0.038**	−0.40 (-0.83, 0.03)	0.068
Food as a reward	0.25 (0.05, 0.44)	**0.01**	0.26 (0.05, 0.48)	0.015	0.28 (0.07, 0.49)	**0.009**
**Physical environment**	**Model 1**		**Model 5**		**Model 6**	
Home availability of energy-dense snack foods	−0.04 (-0.09, 0.01)	0.142	−0.05 (-0.10, 0.01)	0.073		
Home availability of fruit and vegetables	0.15 (0.08, 0.23)	**0.000**	0.11 (0.03, 0.18)	**0.004**	0.08 (0.01, 0.17)	**0.04**
Home accessibility of energy-dense snack foods	−0.16 (-0.28, −0.04)	**0.010**	−0.11 (-0.25, 0.03)	0.117		
Home accessibility of fruit and vegetables	0.11 (0.04, 0.19)	**0.002**	0.11 (0.03, 0.19)	**0.000**	0.04 (-0.04, 0.12)	**0.33**

Bold indicates p < 0.001.

**Table 3:** Regression coefficients and 95% CIs from multiple linear regression analyses: individual, behavioural, social and physical environmental variables and (Model 1) child Vegetable consumption adjusted for child gender and age; (Model 2) child Vegetable consumption adjusted for child gender and age and all ‘individual’ variables significant in the unadjusted linear regression analyses; (Model 3) child Vegetable consumption adjusted for child gender and age and all ‘behavioural’ variables significant in the unadjusted linear regression analyses; (Model 4) child Vegetable consumption adjusted for child gender and age and all ‘social environmental’ variables significant in the unadjusted linear regression analyses; (Model 5) child Vegetable consumption adjusted for child gender and age and all ‘physical environmental’ variables significant in the unadjusted linear regression analyses; (Model 6) child Vegetable consumption adjusted for child gender and age and all variables significant in Models 2–5.

Individual	Vegetable (frequency/day)		Vegetable (frequency/day)		Vegetable (frequency/day)	
	Unstandardised regression coefficient (95% CI)	*P*	Unstandardised regression coefficient (95% CI)	*P*	Unstandardised regression coefficient (95% CI)	*P*
	Model 1		Model 2		Model 6	
Habit for eating snack foods while watching TV	−0.29 (-0.41, −0.16)	**0.000**	−0.25 (-0.39, −0.11)	**0.000**	−0.09 (-0.24, 0.08)	0.251
Habit for eating fruit and vegetables while watching TV	0.18 (0.06, 0.31)	**0.005**	0.18 (0.05, 0.31)	**0.009**	0.07 (-0.07, 0.23)	0.306
Self-efficacy for not eating snack foods when watching TV/DVDs	0.15 (0.03, 0.27)	**0.014**	−0.01 (-0.16, 0.14)	0.870		
Self-efficacy for increasing fruit and vegetable consumption	0.29 (0.17, 0.41)	**0.000**	0.23 (0.10, 0.36)	**0.001**	0.12 (-0.01,0.27)	0.07
Self-efficacy for reducing energy-dense snack food consumption	0.17 (0.05, 0.29)	**0.004**	0.04 (-0.11, 0.19)	0.629		
**Behavioural**	**Model 1**		**Model 3**		**Model 6**	
Eating breakfast while watching TV	−0.08 (-0.37,021)	0.568				
Eating lunch while watching TV	−0.42 (-0.71, −0.13)	**0.005**	−0.17 (-0.59, 0.25)	0.437		
Eating dinner while watching TV	−0.57 (-0.86, −0.28)	**0.000**	−0.52 (-0.93, −0.11)	**0.012**	−0.28 (-0.63, 0.06)	0.100
Eating fruit and vegetables while watching TV	0.74 (0.40,1.09)	**0.000**	1.06 (0.69, 1.42)	**0.000**	0.61 (0.17, 1.05)	**0.007**
Eating energy-dense snacks while watching TV	−0.601 (-0.96, −0.24)	**0.001**	−0.54 (-0.92, −0.16)	**0.006**	−0.07 (-0.57, 0.44)	0.800
**Social environment**	**Model 1**		**Model 4**		**Model 6**	
Eating breakfast at home together with parents	−0.15 (-0.46, 0.16)	0.345				
Eating dinner at home together with parents	0.48 (0.12, 0.85)	**0.009**	0.46 (0.08, 0.85)	**0.019**	0.09 (-0.31, 0.49)	0.653
Eating dinner in front of the TV with parents	−0.51 (-0.84, −0.18)	**0.002**	−0.37 (-0.75, −0.02)	0.065		
Eating breakfast in front of the TV with parents	−0.29 (-0.67, 0.08)	**0.120**	−0.08 (-0.51, 0.35)	0.711		
Eating snacks in front of the TV with parents	−0.36 (-0.71, −0.02)	**0.04**	−0.02 (-0.042, 0.38)	0.919		
Parental pressure to eat	0.15 (-0.13,0.44)	0.269				
Parental food restriction	−0.47 (-0.84, −0.11)	**0.01**	−0.45 (-0.82,-0.09)	**0.016**	−0.30 (-0.71, 0.10)	0.140
Food as a reward	0.04 (-0.14, 0.29)	0.67				
**Physical environment**	**Model 1**		**Model 5**		**Model 6**	
Home availability of energy-dense snack foods	−0.07 (-0.12–0.03)	**0.002**	−0.08 (-0.13, −0.04)	**0.001**	−0.03 (-0.09, 0.03)	0.402
Home availability of fruit and vegetables	0.22 (0.15, 0.28)	**0.000**	0.23 (0.16, 0.30)	**0.000**	0.17 (0.09, 0.25)	**0.000**
Home accessibility of energy-dense snack foods	−0.20 (-0.31, −0.09)	**0.000**	−0.08 (-0.21, 0.05)	0.226		
Home accessibility of fruit and vegetables	0.05 (-0.02, 0.12)	**0.132**	0.03 (-0.04, 0.10)	0.409		

Bold indicates p < 0.001.

**Table 4:** Regression coefficients and 95% CIs from multiple linear regression analyses: individual, behavioural, social and physical environmental variables and (Model 1) child energy-dense snack food consumption adjusted for child gender and age; (Model 2) child energy-dense snack food consumption adjusted for child gender and age and all ‘individual’ variables significant in the unadjusted linear regression analyses; (Model 3) child energy-dense snack food consumption adjusted for child gender and age and all ‘behavioural’ variables significant in the unadjusted linear regression analyses; (Model 4) child energy-dense snack food consumption adjusted for child gender and age and all ‘social environmental’ variables significant in the unadjusted linear regression analyses; (Model 5) child energy-dense snack food consumption adjusted for child gender and age and all ‘physical environmental’ variables significant in the unadjusted linear regression analyses; (Model 6) child energy-dense snack food consumption adjusted for child gender and age and all variables significant in Models 2–5.

Individual	Energy-dense snacks (frequency/day)		Energy-dense snacks (frequency/day)		Energy-dense snacks (frequency/day)	
	Unstandardised regression coefficient (95% CI)	*P*	Unstandardised regression coefficient (95% CI)	*P*	Unstandardised regression coefficient (95% CI)	*P*
	Model 1		Model 2		Model 6	
Habit for eating snack foods while watching TV	1.04 (0.79, 1.29)	**0.000**	0.93 (0.65, 1.18)	**0.000**	0.40 (0.12, 0.68)	**0.005**
Habit for eating fruit and vegetables while watching TV	0.18 (-0.09, 0.45)	**0.183**	−0.13 (-0.40, 0.13)	0.318		
Self-efficacy for not eating snack foods when watching TV/DVD's	−0.40 (-0.65, −0.10)	**0.002**	−0.156 (-0.41, 0.09)	0.205		
Self-efficacy for increasing fruit and vegetable consumption	−0.35 (-0.61, −0.10)	**0.006**	−0.18 (-0.44,0.07)	0.160		
Self-efficacy for reducing energy-dense snack food consumption	−0.09 (-0.34, 017)	0.500				
**Behavioural**	**Model 1**		**Model 3**		**Model 6**	
Eating breakfast while watching TV	0.81 (0.19, 1.43)	**0.010**	0.23 (-0.46, 0.92)	0.508		
Eating lunch while watching TV	1.34 (0.73, 1.95)	**0.000**	0.38 (-0.51, 1.28)	0.397		
Eating dinner while watching TV	0.97 (0.36, 1.59)	**0.002**	−0.21 (-1.02, 0.61)	0.617		
Eating fruit and vegetables while watching TV	0.59 (-0.16, 1.34)	**0.123**	−0.29 (-1.01, 0.43)	0.478		
Eating energy-dense snacks while watching TV	4.08 (3.39, 4.77)	**0.000**	3.84 (3.08, 4.59)	**0.000**	2.03 (1.19, 2.87)	**0.000**
**Social environment**	**Model 1**		**Model 4**		**Model 6**	
Eating breakfast at home together with parents	−0.29 (-0.94, 0.37)	0.395				
Eating dinner at home together with parents	−0.19 (-0.96, 0.59)	0.629				
Eating dinner in front of the TV with parents	1.59 (0.89, 2.28)	**0.000**	0.54 (-0.27, 1.36)	0.187		
Eating breakfast in front of the TV with parents	1.65 (0.87, 2.43)	**0.000**	0.42 (-0.49, 1.32)	0.366		
Eating snacks in front of the TV with parents	2.74 (2.04, 3.44)	**0.000**	2.19 (1.34, 3.04)	**0.000**	0.69 (-0.05,1.45)	0.070
Parental pressure to eat	0.37 (-0.22, 0.95)	**0.222**	0.57 (-0.09, 1.22)	0.089		
Parental food restriction	1.25 (0.47, 2.02)	**0.002**	0.90 (0.06, 1.74)	**0.040**	−0.51 (-1.22, 0.21)	0.164
Food as a reward	0.26 (-0.12,0.64)	**0.184**	0.11 (-0.29, 0.50)	0.605		
**Physical environment**	**Model 1**		**Model 5**		**Model 6**	
Home availability of energy-dense snack foods	0.52 (0.44, 0.60)	**0.000**	0.51 (0.41, 0.60)	**0.000**	0.34 (0.23, 0.0.44)	**0.000**
Home availability of fruit and vegetables	−0.12 (-0.27,0.03)	**0.104**	−0.19 (-0.33, −0.06)	**0.006**	−0.11 (-0.25, 0.02)	0.090
Home accessibility of energy-dense snack foods	0.77 (0.54, 0.99)	**0.000**	0.24 (0.01,0.48)	**0.050**	0.04 (-0.18,0.28)	0.713
Home accessibility of fruit and vegetables	0.11 (-0.03,0.26)	**0.129**	0.05 (-0.09, 0.18)	0.478		

Bold indicates p < 0.001.
